# Molecular characterization and demographic insights into soybean bud borer (Lepidoptera: Tortricidae) in Brazil

**DOI:** 10.1093/jisesa/ieae019

**Published:** 2024-03-08

**Authors:** Davi de Souza Fernandes, Renato Jun Horikoshi, Patrick M Dourado, Ramiro F L Ovejero, Geraldo U Berger, Marcoandre Savaris, John W Brown, Alberto Soares Corrêa

**Affiliations:** Department of Entomology and Acarology, “Luiz de Queiroz” College of Agriculture, University of São Paulo (ESALQ/USP), Piracicaba, São Paulo, Brazil; Department of Entomology and Acarology, “Luiz de Queiroz” College of Agriculture, University of São Paulo (ESALQ/USP), Piracicaba, São Paulo, Brazil; Department of Regulatory Science, Bayer Crop Science Brazil, São Paulo, São Paulo, Brazil; Department of Regulatory Science, Bayer Crop Science Brazil, São Paulo, São Paulo, Brazil; Department of Regulatory Science, Bayer Crop Science Brazil, São Paulo, São Paulo, Brazil; Department of Regulatory Science, Bayer Crop Science Brazil, São Paulo, São Paulo, Brazil; Department of Entomology and Acarology, “Luiz de Queiroz” College of Agriculture, University of São Paulo (ESALQ/USP), Piracicaba, São Paulo, Brazil; Department of Entomology, National Museum of Natural History, Smithsonian Institution, Washington, DC, USA; Department of Entomology and Acarology, “Luiz de Queiroz” College of Agriculture, University of São Paulo (ESALQ/USP), Piracicaba, São Paulo, Brazil

**Keywords:** *Crocidosema aporema*, damage, host plant, insect pest, DNA barcode

## Abstract

The soybean bud borer, a soybean pest in Brazil, was initially identified as *Crocidosema aporema* (Walsingham 1914) (Lepidoptera: Tortricidae). Outbreaks of this species have recently increased, but identification of this pest remains uncertain, and the historical factors associated with its geographic distribution in Brazil are little known. Here, we conducted a species characterization and phylogeographic analysis based on molecular and morphological evidence. Ninety individuals of bud-borers Lepidoptera were collected in different regions of Brazil. We sequenced COI and COII mitochondrial genes and examined wing patterns and male genital morphology. DNA barcoding approach revealed that 10 individuals were *Argyrotaenia sphaleropa* (Meyrick 1909) (Lepidoptera: Tortricidae) and 80 were a species of the genus *Crocidosema* Zeller. The morphology of the adult genitalia and wings proved to be insufficient to confirm the identification of Brazilian individuals as *C. aporema*, a species originally described from a high-elevation site in Costa Rica. Furthermore, the genetic distance between putative *C. aporema* specimens from Brazil and Costa Rica (ranging from 5.2% to 6.4%) supports the hypothesis that the Brazilian specimens are not referable to *C. aporema.* Our analysis revealed a single genetic strain (i.e., species) with low genetic diversity on soybean crops. We found no indication that the genetic structure was related to geographic distance among populations or edaphoclimatic regions. The population expansion of the soybean bud borer coincides with the increase in the area of soybean production in Brazil, suggesting that expanded soybean farming has allowed a significant increase in the effective population size of this pest.

## Introduction

The bud borer, *Crocidosema aporema* (Walsingham 1914) (Lepidoptera: Tortricidae), is an oligophagous pest of Fabaceae in the Americas, reported from Argentina, Brazil, Chile, Colombia, Costa Rica, Peru, and Uruguay (Biezanko 1961, Iede 1980, Pereyra and Sanchez 1998). Although the presence of the species is not confirmed in North America, it is intercepted at US ports of entry and has the potential to become an invasive species there (Brown 2011, Gilligan and Passoa 2014). Larval feeding results in damage to the tips, buds, and leaf axils of its host plants (Corrêa and Smith 1976). A soybean bud borer initially identified as *C. aporema* was found in Brazil in about 1960 (Biezanko 1961). Subsequent reports of this species have been sporadic and always associated with damage to soybeans in southern Brazil (Corrêa and Smith 1976, Guillen 1977, Calderon and Foerster 1979, Gazzoni and De Oliveira 1979). After the 1960s, this species attracted less attention, partly due to its minor economic importance compared to other soybean defoliators (Foerster et al. 1983, Horikoshi et al. 2021a).

In recent years, the distribution and abundance of certain species have changed considerably with the emergence of new native and invasive pests (Veres et al. 2013, Madden et al. 2019, Walter et al. 2020, Horikoshi et al. 2021a, Yang et al. 2022). These changes in geographic distribution, frequency of population outbreaks, and host range have raised doubts about some species identifications, resulting in delays in implementing effective pest-control strategies. Such is the current scenario for the soybean bud borer, with recent reports of population outbreaks on Cry1Ac Bt soybean and non-Bt soybean and a range expansion from southern to central South America (Horikoshi et al. 2021b, Hayashida et al. 2023).

The correct identification of *C. aporema* is hampered by questions of synonymy and the presence of undescribed species of *Crocidosema* in the Americas (Razowski and Becker 2014). Additionally, other species of Tortricidae, such as *Argyrotaenia sphaleropa* (Meyrick 1909) (Lepidoptera: Tortricidae), have also been reported in soybean fields in Brazil (Neves and Silva 2021). These 2 facts raise doubts about whether the ‘soybean bud borer’ responsible for damaging soybean crops in different Brazilian regions is truly *C. aporema*.

Molecular markers, a valuable tool for species characterization, are highly useful in monitoring agricultural pests and detecting invasive insects (Hodgetts et al. 2016, van Klink et al. 2022). These molecular markers are even more helpful when a species’ morphological identification is uncertain, and when early life stages, which are notoriously difficult to identify based on morphology, are easily sampled in the field (De Barro and Ahmed 2011, Parish et al. 2017, Kapantaidaki et al. 2022, Yan et al. 2022). For these and other purposes, the mitochondrial gene cytochrome c oxidase subunit I (*COI*) is the most widely used marker (Hebert et al. 2003). Furthermore, COI and other mitochondrial genes provide information on speciation processes and population demographic history because they are sensitive to genetic drift and have a low recombination rate due to exclusive maternal inheritance (Avise et al. 1987, Neiman and Taylor 2009, Choupina and Martins 2015).

Phylogeographic studies can provide insight into the historical demography of the soybean bud borer in Brazil and how its populations are distributed in geographic space. They can be used to determine whether populations recently reported in midwestern Brazil are the same as those in southern Brazil, whether these populations have recently expanded, and whether they are localized or form a single homogeneous population distributed throughout the country. Therefore, the objectives of the present study were (i) to confirm that *C. aporema* is the main bud borer species associated with soybean crops in Brazil; (ii) to estimate the genetic diversity, population structure, and demographic history of this species in the country; and (iii) to infer the temporal variation in effective population size of this species associated with soybean fields in Brazil. The answers to these questions can contribute to managing this resurgent agricultural pest in Brazil.

## Materials and Methods

### Insect Collection and Morphological Identification

**Fig. 1.**
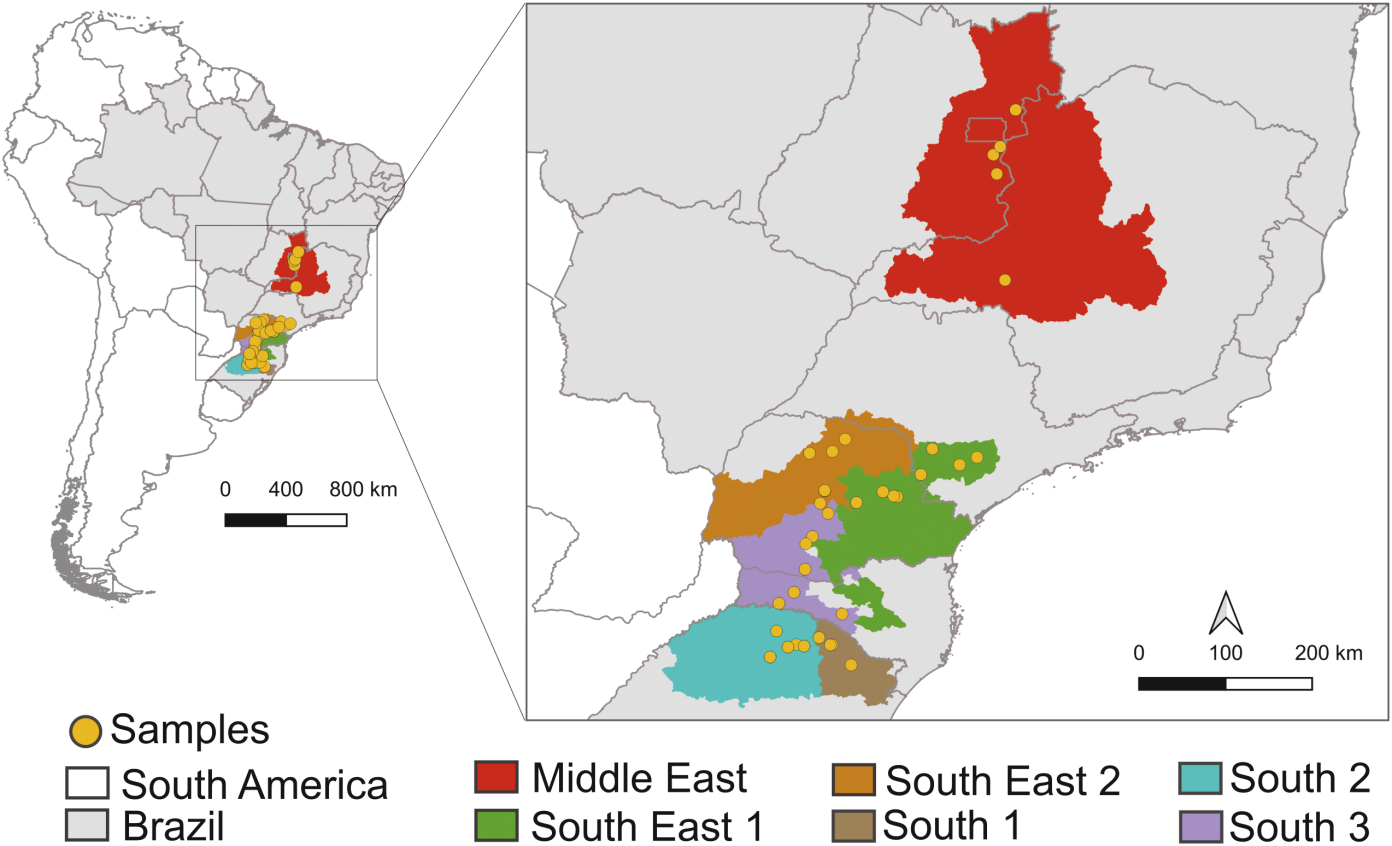
Distribution of bud borer individuals collected in Brazil. The samples were grouped based on edaphoclimatic regions: South (1, 2, 3), Southeast (1, 2), and Midwest.

Bud borer larvae were collected from 2020 through 2022 from 38 soybean fields (SISBIO licenses # 61826 and 61824) (see Supplementary Table S1). The collections were divided into 6 groups (South 1, South 2, South 3, Southeast 1, Southeast 2, and Midwest) based on edaphoclimatic Brazilian regions (IBGE), soybean macro-regions (Kaster and Farias 2012), and geographic distance between samples (Fig. 1). Most larvae were preserved in vials with 100% ethanol and stored at −20 °C.

Some larvae were reared to the adult stage under laboratory conditions: 25 °C, 70% relative humidity, 14:10 L:D, and a semi-artificial diet (Greene et al. 1976) (Supplementary Table S1). Laboratory-reared adults were characterized morphologically, following descriptions and illustrations by Gilligan and Passoa (2014), Gilligan and Epstein (2014) (see Supplementary Figs. S1–S3). Adults and their genitalia were photographed using a Leica Digital DFC450 camera, integrated into a Leica MDG41 system (Leica Microsystems, Heerbrugg, Switzerland). Wings of adults from Capão Bonito, the state of Rio Grande do Sul, and genitalia from adults collected at 5 different sites throughout Brazil were mounted on microslides. The specimens were deposited in the collection of the Museu de Entomologia ‘Luiz de Queiroz’ (MELQ), Departamento de Entomologia e Acarologia, Escola Superior de Agricultura ‘Luiz de Queiroz’, Piracicaba, São Paulo, Brazil (numbers ESALQENT1755-72). The same specimens used for genitalia examination were used for molecular analysis.

### DNA Extraction, PCR, and Sequencing

DNA extraction was performed with a CTAB and proteinase K-based protocol (Doyle and Doyle 1987) for both larvae and adults. The cytochrome c oxidase subunit I (COI) and cytochrome c oxidase subunit II (COII) gene fragments were amplified using the universal primers LCO1490 and HCO2198 (Folmer et al. 1994), and Patrick and Eva for COI and COII (Simon et al. 1994), respectively. The PCR reactions were performed in a total volume of 25 µl, containing 11.2 µl of ultrapure H_2_O, 2.5 µl of PCR buffer 10× (Invitrogen), 2 µl MgCl_2_ (50 mM), 2 µl dNTP (2.5 mM), 2 µl of each primer (F and R), 0.3 µl of Taq DNA Polymerase (Synapse Inc.), and 3 µl of DNA for both COI and COII. The amplification reaction consisted of an initial denaturation step at 95 °C for 3 min, followed by 28 cycles at 94 °C for 35 s, 53 °C for 45 s, 72 °C for 2 min, and a final extension at 72 °C for 10 min. The PCR products were submitted for Sanger sequencing at the Animal Biotechnology Laboratory (USP/ESALQ) in Brazil.

### Sequence Editing and Assembly

Sequences were assessed by manual analysis of chromatograms, which involved the removal of primer regions and sequence tips, using Benchling [Biology Software, https://www.benchling.com/]. NUMTs (nuclear introns in the mitochondrial genome) were examined, and none was identified using MEGAX (Kumar et al. 2018). A total of 90 sequences of COI (658 bp) were obtained, along with 90 sequences of COII (630 bp). For the BLAST search and phylogenetic analysis, only COI sequences were used. Genetic diversity and demographic analyses were performed using concatenated COI and COII sequences of the same individuals.

### Data Analysis

#### DNA barcoding

The 90 COI sequences were compared with those on the BOLDSystem database with the ‘Species Level Barcode Records’ selection for species identification (Ratnasingham and Hebert 2007). To our COI dataset, we added 18 additional sequences of *C. aporema* from Costa Rica and 30 sequences of other *Crocidosema* species available on the BOLDSystem website, using the “Species Level Barcode Records” (Ratnasingham and Hebert 2007) (Supplementary Table S2). The pairwise genetic distances (K2-P) among *Crocidosema* spp. were calculated using MEGA X software (Kumar et al. 2018). Additionally, we built a Bayesian phylogenetic tree with COI gene sequences (Fig. 1). The phylogenetic analysis included 2 main steps: first, we found the best nucleotide substitution model, GTR+G+I, using the software MRMODELTEST v2.3 in PAUP (Nylander 2004). Second, the Bayesian phylogenetic tree was built in MrBayes v3.1.2 (Ronquist and Huelsenbeck 2003) using 2 simultaneous runs of 10 million generations each, with 1 cold and 3 heated chains in each run. The first 25% of the trees were discarded as burn-in samples, and the consensus tree of the 2 independent runs was obtained with posterior probabilities >0.50.

#### Haplotype network, genetic diversity, and AMOVA among Brazilian individuals

For our analyses, 80 specimens of Brazilian *Crocidosema* sp. were divided into 6 groups (South 1, South 2, South 3, Southeast 1, Southeast 2, and Midwest) as previously described. All analyses were conducted with concatenated COI + COII gene fragments (=1,288 bp).

The genealogical relationship among haplotypes was estimated using a median-joining network haplotype in PopArt (Leigh and Bryant 2019). The number of haplotypes (n), haplotype diversity (Hd), nucleotide diversity (π), number of polymorphic sites (S), and mean nucleotide differences by sequences (k) for each group were estimated in DNAsp v5 (Rozas et al. 2017).

The analysis of molecular variance (AMOVA) with parametric bootstrap (1,000 replicates) and 5% significance level to investigate the presence of genetic structure among *Crocidosema* groups was calculated in Arlequin v.3.5 (Excoffier and Lischer 2010).

#### Demographic analysis

We calculated Tajima’s D (Tajima 1989) and Fu’s Fs (Fu 1997) neutrality tests to identify *Crocidosema* populations that were expanding, contracting, or in equilibrium, using ARLEQUIN v.3.5 (Excoffier and Lischer 2010). The analyses were performed with 1,000 permutations, with coalescence simulations, statistical significance at a confidence interval of 95%, and a *P*-value less than 0.05 for Tajima and 0.02 for Fu’s Fs test (Excoffier and Lischer 2010).

Afterward, we performed the mismatch distribution analysis using the indices sum of squared deviations (SSD) and the Raggedness index (*r*), comparing them to a *P*-value, which indicates the goodness-of-fit for the population expansion model. The SSD test assumes the null hypothesis of spatial population expansion. In contrast, the *r* raggedness test reveals whether a population exhibits a unimodal expansion, characterized by a recent and intense expansion, or a multimodal expansion, marked by different expansion events in evolutionary time. In both tests, a nonsignificant value converges to a population expansion.

#### Bayesian skyline plot

The historical demography of the soybean bud borer population was reconstructed using the Bayesian Relaxed Skyline Plot (Ho and Shapiro 2011). For this step, the concatenated COI + COII haplotypes were used without considering any hierarchical level and population structure, because it was not found. Thus, the relative age of haplotype divergence was estimated by concatenated mtDNA genes in BEAST v.1.8.4. The substitution model was determined by PARTITIONFINDER v.1.1.1 to be HKY. The substitution rate was estimated using a strict molecular clock and coalescent tree before a normal population size distribution. At the same time, other parameters were set to default values in the software and gamma distribution. The molecular clock was calibrated based on the insect mitochondrial genome, which has a nucleotide divergence rate of 3.54% per million years, equivalent to 0.0177 after accounting for the haploid nature of the mitochondrial genome (Papadopoulou et al. 2010). The parameters were set using BEAUTY for 150 million generations. The quality of the coalescent simulation model was assessed using TRACER v.1.6. The resulting model was accepted since it showed effective sample sizes greater than 200, with the mean calculated and 95% posterior density interval (HPD) for the time of divergence.

## Results

### Species Identification

Males of most species of *Crocidosema* can be separated based on unique combinations of conspicuous male secondary structures (e.g., specialized wing scales, costal folds, hairpencils, etc.), even though features of the male genitalia may be similar. Females exhibit no such conspicuous differences in secondary structures or genitalia, the latter being extremely similar among many species. Because the holotype specimen of *C. aporema*, housed in the Natural History Museum, London, is a female from Costa Rica, its association with any particular male is equivocal. Hence, morphology is of limited value in determining the ‘true’ identity of *C. aporema*.

Although DNA barcodes are highly useful for associating conspecific males and females, DNA barcodes are unavailable for the holotype of *C. aporema*, which is old (Walsingham 1914). DNA barcodes recognize at least 5 species of *Crocidosema* in Costa Rica alone, which does not exclude the possibility of unequivocally associating any of these males with the holotype female. To clearly characterize the morphology of the soybean borer present in Brazil, we provide images of the body, wings, and male genitalia (see Supplementary Figs. S2 and S3).

### Molecular Characterization

Among 90 COI sequences compared in the BOLDSystem, 10 individuals were unambiguously identified as *Argyrotaenia sphaleropa* (Meyrick 1909) (Lepidoptera: Tortricidae) (GenBank number: PP212812-PP212821), with 100% similarity to specimens in BOLDSystems. The other 80 COI sequences (COI GenBank number: PP175436-PP175515) submitted to BOLDSystems showed genetic similarities with *C. aporema*, *C. accessa* (Heinrich 1931), and *C. perplexana* (Fernald 1901), from 94.44% to 93.07%.

**Fig. 2.**
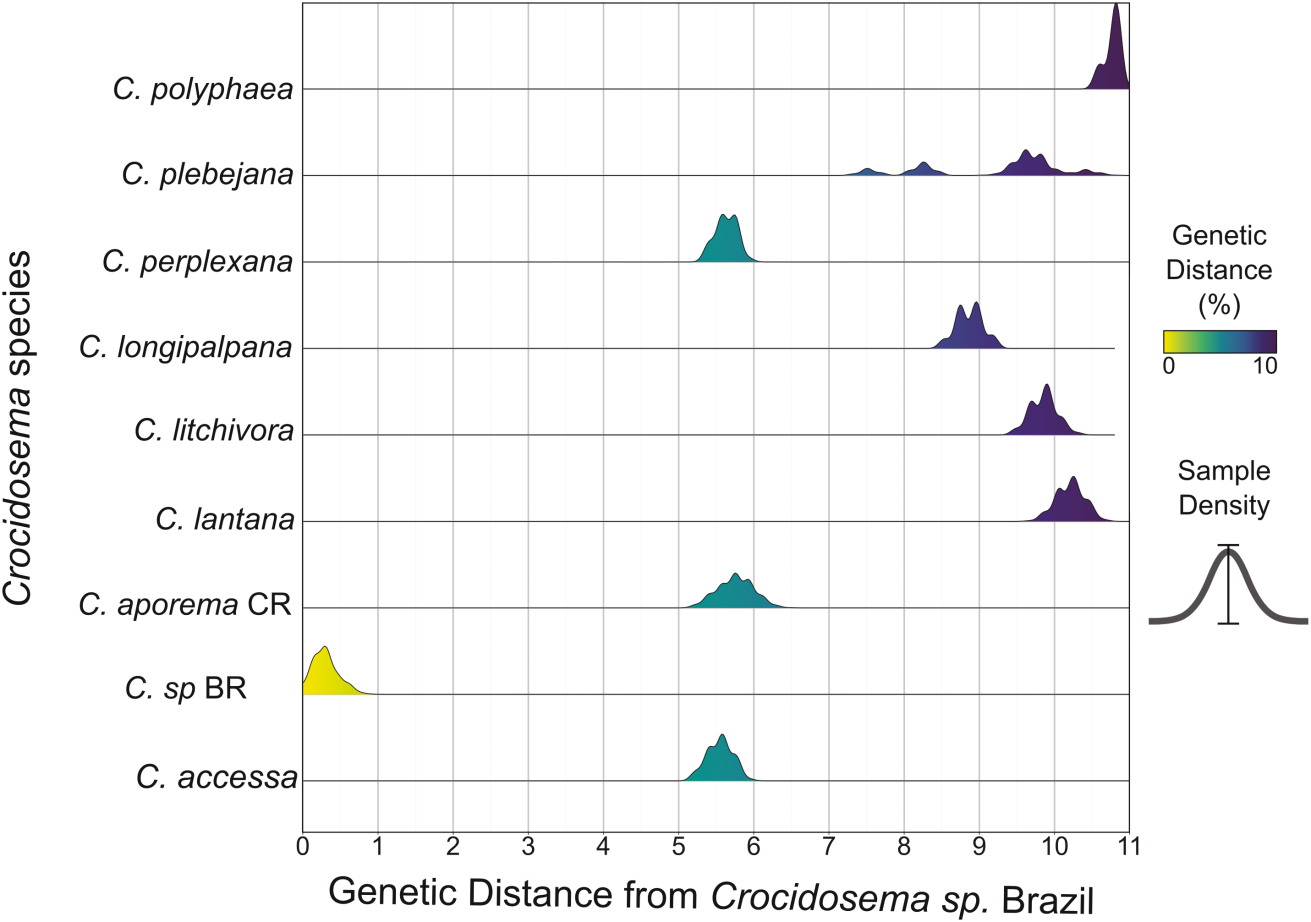
Genetic distance between cytochrome c oxidase subunit I (COI) haplotypes of *Crocidosema* spp. from Brazil and the database BOLDSystem (Ratnasingham and Hebert 2007). For the corresponding COI sequence codes, see Supplementary Table S2.

The genetic distances among 80 Brazilian *Crocidosema* individuals, including those identified using morphological features, ranged from 0.00% to 0.9% (Fig. 2). The genetic distances (K2-P) between Brazilian *Crocidosema* individuals and Costa Rican ‘*C. aporema*’ ranged from 5.2% to 6.4%. Similar genetic distances were observed between Brazilian *Crocidosema* individuals and *C. perplexana* and *C. accessa* (Fig. 2). The genetic distances between Brazilian *Crocidosema* individuals and other *Crocidosema* species were higher than 7.2% (Fig. 2).

**Fig. 3.**
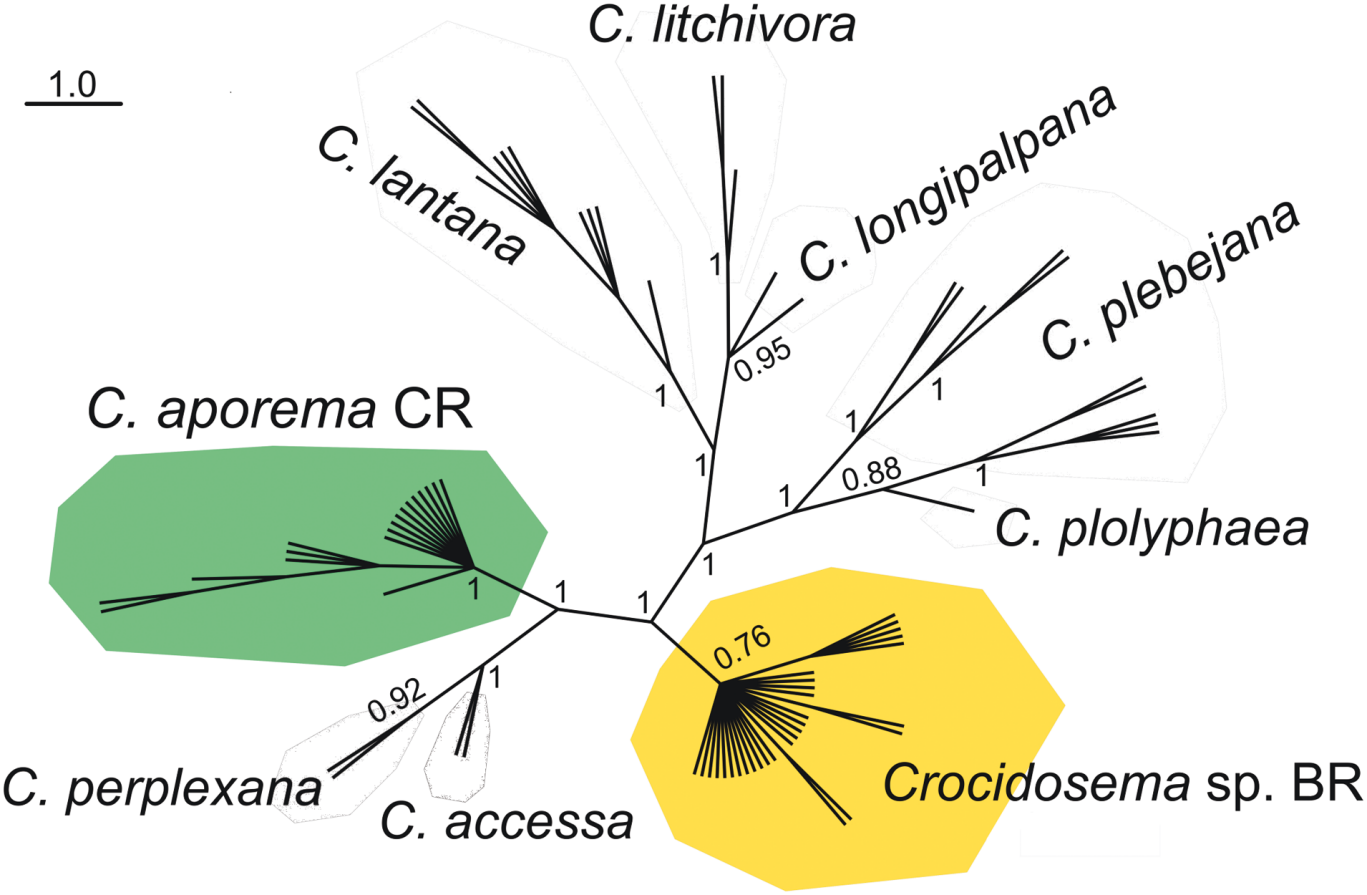
Bayesian Phylogenetic tree of *Crocidosema* species based on cytochrome c oxidase subunit I (COI) haplotypes of *Crocidosema* spp. from Brazil and the database BOLDSystem (Ratnasingham and Hebert 2007). For the corresponding COI sequence codes, see Supplementary Table S2.

The Bayesian tree based on Brazilian *Crocidosema* individuals and other *Crocidosema* species obtained from BOLDSystems indicated that individuals from Brazil and Costa Rica identified as *C. aporema* formed a paraphyletic group closely associated with *C. perplexana* and *C. accessa* (Fig. 3). No Brazilian specimens linked at the typical species level (less than 2%) with specimens from elsewhere. Apparently, the taxon *C. aporema* consists of a complex of closely related species (cryptic species) in the Americas.

### Genetic Diversity and Population Structure

**Fig. 4.**
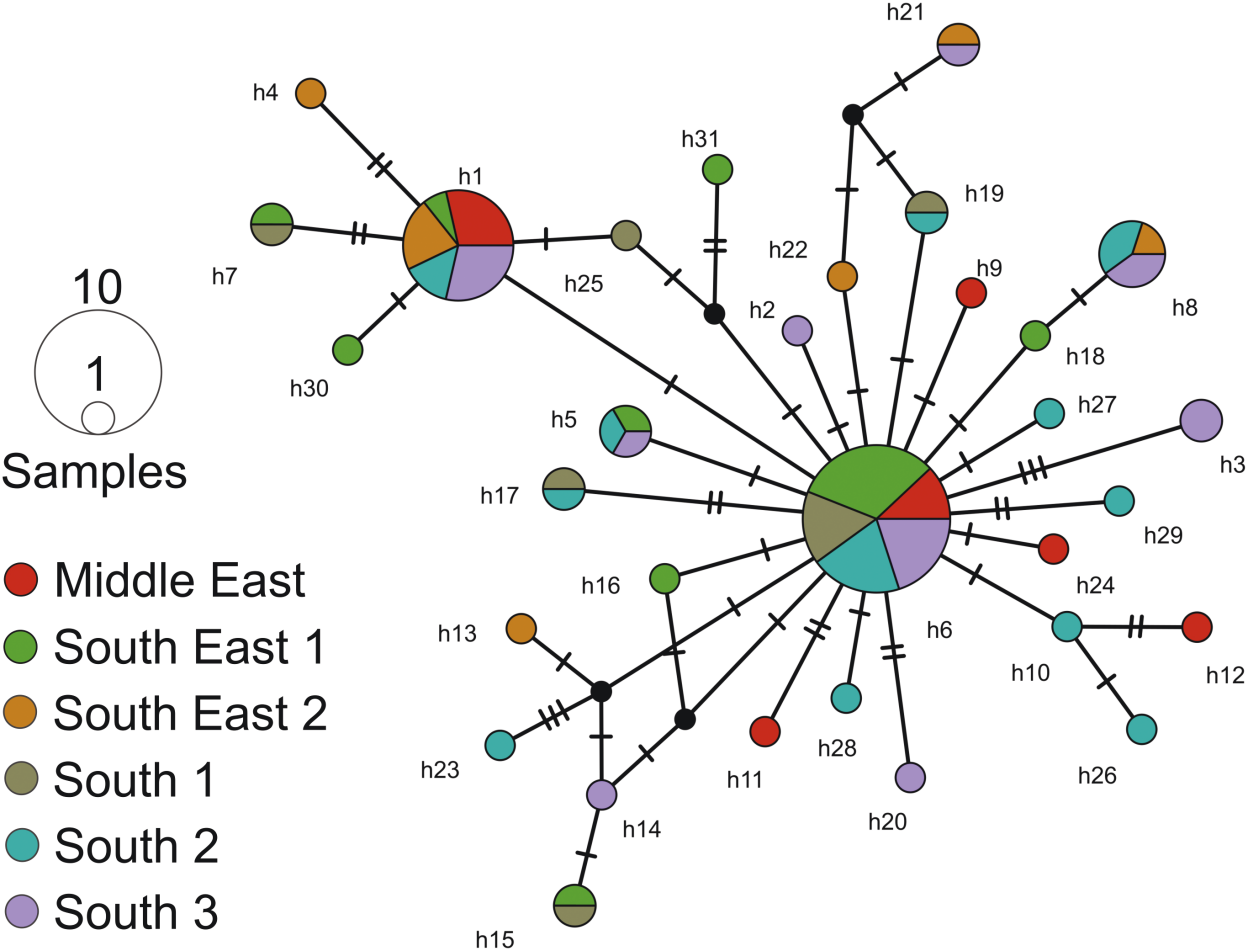
Median-joining network haplotype of *Crocidosema* sp. from Brazil, based on cytochrome c oxidase subunit I (COI) and cytochrome c oxidase subunit II (COII) sequence fragments.

The network analysis revealed a recent genealogical relationship among the 31 soybean bud borer haplotypes based on COI-COII (COII Genbank number: PP209862-PP209941), with few mutation steps separating the haplotypes (Fig. 4). The network was star-shaped, with 2 more-frequent haplotypes H1 (*n* = 11) and H6 (*n* = 18), and other haplotypes at lower frequencies (*n* < 5).

DNA barcodes of soybean bud borers from Brazil revealed an overall haplotype diversity, nucleotide diversity, and a mean number of nucleotide differences of Hd = 0.870, π = 0.0017, and *k* = 2.22, respectively (Table 1). The highest haplotype diversity was found in the ‘South 2’ (Hd = 0.922) region, and the lowest diversity in ‘Southeast1’ (Hd = 0.767). The nucleotide diversity was high for ‘Southeast 2’ (π = 0.0024), and low for ‘Midwest’ (π = 0.0013).

**Table 1.** Diversity indexes for Brazilian *Crocidosema* sp. based on concatenated COI and COII gene fragments. Number of insects, number of haplotype (*H*), number of polymorphic sites (*S*), haplotype diversity (*Hd*), nucleotide diversity (π), and the mean number of polymorphic differences (*k*) per group

Group	Number of insects	*H*	*S*	*hd*	Π	*k*
Middle West	11	6	8	0.836	0.0013	1.78
Southeast 1	16	9	12	0.767	0.0014	1.81
Southeast 2	8	6	10	0.893	0.0024	3.00
South 1	9	6	10	0.833	0.0018	2.38
South 2	18	12	16	0.922	0.0017	2.25
South 3	18	9	15	0.882	0.0018	2.41
Brazil	80	31	40	0.870	0.0017	2.22

Further, the AMOVA revealed no statistical significance for the population genetic structure of soybean bud borers (Φ_ST_ = 0.019; *P* = 0.30), confirming that there are no subgroups associated with distances among populations or different soybean regions (Table 2).

**Table 2.** Analysis of molecular variance (AMOVA) for the genetic structure of Brazilian *Crocidosema* sp. populations based on concatenated COI and COII gene fragments

Source of variation	*g.l.*	Sum of squares	Percentage of variance	Fixation index (*P-*value)
Among population	33	36.00	1.96	Fst = 0.019 (*P* = *0.30*)
Within Population	46	47.95	98.04	
Total	79	83.96		

### Demographic Indexes

Tajima’s D neutrality test and Fu’s Fs neutrality test yielded significant negative values, with *P* < 0.05 and *P* < 0.02, respectively, indicating a recent population expansion or purifying selection, with an excess of low-frequency polymorphisms in soybean bud borer populations from Brazil (Table 3). On the other hand, regarding specific clusters, only the Southeast 1 and South 2 regions showed population expansion for the indices. The SSD mismatch analysis did not yield significant values (*P* > 0.05), supporting the null hypothesis of demographic expansion. Similarly, the raggedness indices supported a spatial expansion model of the populations in a unimodal way (Table 3). Finally, all clusters showed similarity in the calculated Tau (τ) values, where the 95% confidence interval did not indicate differences between groups and the total population for the age of population expansion, reinforcing the idea of a recent demographic expansion (Table 3).

**Table 3.** Demographic history for Brazilian *Crocidosema* sp. based on concatenated COI and COII gene fragments

Groups	*N*	Tajima’s *D*	*Fu’s* Fs	SSD (*P*-value)	*r* (*P*-value)	τ (α = 95%)
Middle West	11	−1.38	−1.81	0.008 (0.60)	0.069 (0.60)	1.4 (0.0–3.3)
Southeast 1	16	−2.12*	−5.19**	0.007 (0.50)	0.054 (0.50)	0.8 (0.9–3.8)
Southeast 2	8	−1.16	−1.42	0.032 (0.50)	0.139 (0.40)	3.6 (2.0–5.3)
South 1	9	−1.82	−2.17	0.001 (0.90)	0.027 (0.90)	2.3 (0.7–4.5)
South 2	18	−1.89	−8.52**	0.007 (0.10)	0.075 (0.07)	2.3 (1.4–3.1)
South 3	18	−1.54	−2.94	0.008 (0.30)	0.037 (0.50)	2.0 (1.6–4.1)
Brazil	80	−2.36*	−27.19**	0.005 (0.75)	0.036 (0.34)	2.08 (0.6–4.9)

*P < 0.05 and **P < 0.02.

### Bayesian Skyline Plot

**Fig. 5.**
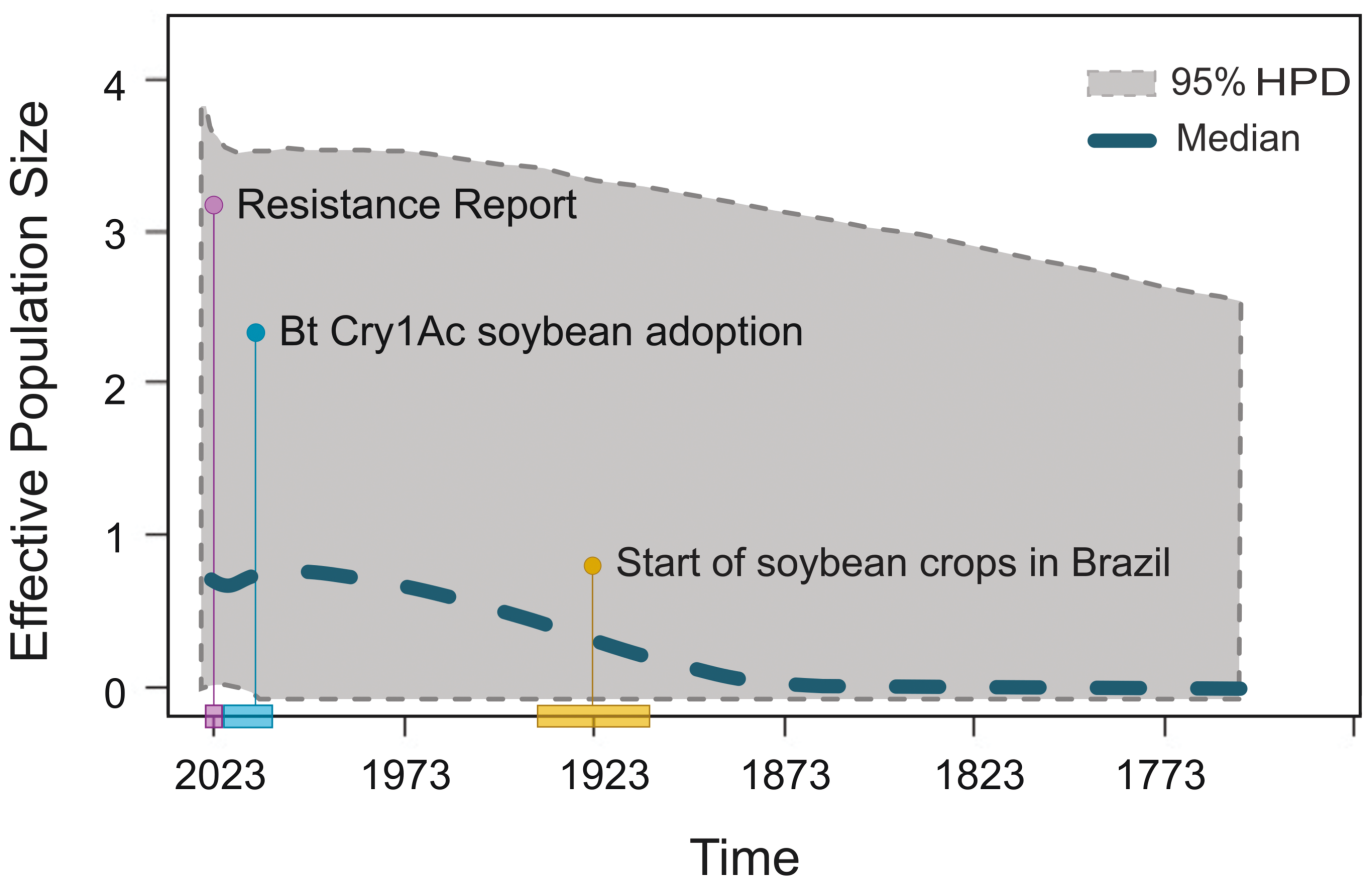
Estimated population dynamics over the last 250 yr for the Brazilian population of *Crocidosema* sp., using the Extended Bayesian Skyline Plot (EBSP) with Population Expansion Time (Time) and Estimated Effective Population Size (Ne), with a 95% highest posterior density (HPD) interval based on cytochrome c oxidase subunit I (COI) and cytochrome c oxidase subunit II (COII) sequence fragments. Vertical bars indicate specific periods: the beginning of soybean cultivation around 1914; the first outbreaks recorded for the population of *Crocidosema* sp. in Brazil in 1960 (Biezanko 1961); and the adoption begin of Bt soybean technology in 2013.

The Bayesian Skyline Plot analysis confirmed that the observed population expansion occurred very recently, starting within the last 150 yr (Fig. 5). The increased rate of population growth of the soybean bud borer coincides with the beginning of soybean cultivation in southern Brazil in the early 20th century (Sousa and Bush 1998). The population expansion continued until around 2010, when the growth stabilized at close to zero, followed by an inflection point in the curve, with a decrease in population size. Finally, in the last 3 yr, the curve reveals a resumption of the increase in effective population size (Fig. 5).

## Discussion

The recent increased attention to the soybean bud borer by soybean producers in Brazil has been driven by more-frequent population outbreaks and control failures of Cry1Ac Bt soybean in various regions over the last few years (Horikoshi et al. 2021b). Our studies revealed the presence of 2 species associated with damage to soybean terminal buds and shoots, *Argyrotaenia sphaleropa* and *Crocidosema* sp.

From our samples, we identified 10 individuals of *A. sphaleropa*, which is typically a leaf-roller rather than a bud borer. However, this species attacks soybean terminal buds, and its larval stages are easily confused with soybean bud borer larvae in the field owing to morphological similarities. *Argyrotaenia sphaleropa* is a polyphagous species that is a common pest of orchards and ornamental plants, including orange (*Citrus sinensis* L.), grape (*Vitis vinifera* L.), tomato (*Solanum lycopersicum* L.), and potato (*S. tuberosum* L.) in Brazil (Biezanko 1961, Neves and Silva 2021).

The low genetic distance among all Brazilian soybean bud borer individuals confirms the presence of a single species of *Crocidosema* in soybean fields in the country. However, the morphological and DNA barcoding analysis provides evidence that the predominant tortricid species in soybean fields is not *C. aporema*. This is reinforced by the fact that the type specimen of *C. aporema* was collected at Volcán de Irazú, a high-altitude area (around 3,000 m) in the Central Cordillera from Costa Rica. Hence, it is unlikely that it is conspecific with lowland species found in agricultural areas. Thus, we conclude that the reports of *C. aporema* in Brazil are probably based on misidentifications stemming from a lack of information about this genus in the country (Corrêa and Smith 1976, Foerster et al. 1983, Altesor et al. 2010, Almeida and Penteado-Dias 2022, Hayashida et al. 2023).

Our phylogeographic analysis revealed moderate mtDNA diversity, an excess of low-frequency haplotypes, and an absence of population genetic structure among the 6 subpopulations of the soybean bud borer that we examined. These results indicate that a single lineage with high genetic similarity among mtDNA haplotypes occurs throughout soybean fields in southern and central Brazil. Thus, despite historical records indicating that soybean bud borer populations were more abundant in southern Brazil (Biezanko 1961, Iede 1980), our phylogeographic data do not support a directional spatial expansion of this species from southern to central Brazil.

The star-shaped haplotype network and the demographic indices confirm the population expansion (increased effective population size) of this species in all subregions sampled. Similar population expansion has been reported for other native lepidopteran soybean pests in Brazil, such as *Chrysodeixis includens* (Walker 1858) (Lepidoptera: Noctuidae) (Silva et al. 2020) and *Rachiplusia nu* (Guenée 1852) (Lepidoptera: Noctuidae) (Perini et al. 2021, Decker‐Franco et al. 2023). This can be explained, in part, by the increase in the availability of soybeans (a non-native host), owing to the continued expansion of soybean cultivation in Brazil in recent decades.

Monoculture agricultural systems create unstable landscapes by alternating between periods of resource abundance and scarcity (Mitchell et al. 2014, Santos et al. 2017). As a result, subpopulations of soybean bud borers residing in agricultural landscapes experience intense colonization and growth, followed by a drastic reduction in population density when crops are unavailable in the field (Santos et al. 2017, Walter et al. 2020). This recolonization process can lead to intense genetic drift, resulting in a reduction of nucleotide diversity, an increase in the number of low-frequency haplotypes, and the dominance of a few haplotypes that quickly spread through the population (Rogers and Harpending 1992, Neiman and Taylor 2009). In the case of this *Crocidosema* species, the short life cycle and the cultivation of Fabaceae in the winter crop seasons may facilitate the rapid recolonization by this pest in soybean fields and with a rapid increase in population density (Corrêa and Smith 1976, Foerster et al. 1983).

The Bayesian Skyline Plot revealed that the temporal population expansion of the soybean bud borer coincided with the increase in cultivated area and geographic expansion of soybeans in Brazil. Initially, soybean fields were located predominantly in the country’s southern region until approximately 1970, when they spread to the central region from Brazil. Interestingly, injury caused by soybean bud borers and high densities of the pest began to be reported in the southern region around 1970 (Corrêa and Smith 1976, Calderon and Foerster 1979), and the time estimate for the increase in the species’ population size overlaps with this period. The BSP analysis also showed a reduction in population size over the last 10 yr. Thus, we can speculate about an association between the beginning of transgenic Bt soybean cultivation in 2013, which initially caused high mortality in soybean bud borer individuals, and the decreasing population demographic expansion associated with the high adoption of Bt soybeans, since more than 85% of soybean areas in Brazil cultivated Bt soybeans in the 2020–2023 seasons (https://www.kynetec.com/significant-growth-recorded-in-brazilian-soybean-nutrition-market). However, the low susceptibility of soybean bud borer populations to Cry1Ac Bt soybean has been observed since 2020 (Horikoshi et al. 2021b), which also overlaps with the resumption of population increases of this species in the BSP analysis.

In conclusion, the low genetic divergence among Brazilian *Crocidosema* individuals confirms the presence of a single soybean bud borer species in Brazil. Comparison among DNA barcodes indicated that the *Crocidosema* species in Brazil is not *C. aporema* but is likely an undescribed species. The fact that it is an undescribed species does not alter conclusions from previous research on this pest since we did not find any other species of the genus *Crocidosema* in soybean areas in Brazil. Even though the development of pest-control methods must consider the species and population diversity before its implementation (Pélissié et al. 2018, Corrêa et al. 2019), information on this species in Brazil remains extremely relevant to its biology and control. Finally, the Brazilian soybean bud borer populations do not exhibit a geographic population structure. In recent years, these populations have undergone a demographic expansion, strongly influenced by the increased cultivation of soybeans and pest management practices (primarily Bt crops). This confirms the considerable impact that agricultural landscapes (e.g., monocultures) and pest management strategies can have on the demography of a native species.

## Supplementary Material

ieae019_suppl_Supplementary_Tables_S1-S2_Figures_S1-S3
