# Supplementary material for: Molecular characterization and demographic insights into soybean bud borer (Lepidoptera: Tortricidae) in Brazil
Source: J Insect Sci. 2024 Mar 8;24(2):5. doi: 10.1093/jisesa/ieae019 (PMC10922366; doi:10.1093/jisesa/ieae019)
Supplement: ieae019_suppl_Supplementary_Tables_S1-S2_Figures_S1-S3 [file ieae019_suppl_supplementary_tables_s1-s2_figures_s1-s3.docx]

**Supplementary material**

**Table S1.** Collecting sample’s locations. City, State, Coordinates, Regional Groups, Number of identified insects by COI analysis, Species identified, Number of *Crocidosema* sp. haplotypes, Haplotypes found.

| **Local** | **UF** | **Latitude** | **Longitude** | **Group** | **Species** | ***H*** | **Haplotypes** |
| --- | --- | --- | --- | --- | --- | --- | --- |
| Cristalina | GO | -16.8326 | -47.5403 | CO | *Crocidosema* sp. | 2 | H1, H9 |
| Cristalina* | GO | -16.1780 | -47.4572 | CO | *Crocidosema* sp. | 5 | H1, H6, H11, H12, H24 |
| Cristalina | GO | -16.3731 | -47.6228 | CO | *Crocidosema* sp. | 1 | H1 |
| Formosa | GO | -15.2982 | -47.0890 | CO | *Crocidosema* sp. | 1 | H6 |
| Perdizes* | MG | -19.3732 | -47.3423 | CO | *Crocidosema* sp. | 2 | H1, H6 |
| Arapongas | PR | -23.4758 | -51.4784 | SU 3 | *Crocidosema* sp. | 1 | H13 |
| Boa Ventura do São Roque | PR | -24.9599 | -51.5814 | SU 3 | *Crocidosema* sp. | 2 | H8, H14 |
| Candói | PR | -24.9599 | -51.5814 | SU 3 | *Crocidosema* sp. | 4 | H1, H2, H3(2) |
| Coronel Domingos Soares | PR | -26,2956 | -52,1332 | SU 3 | *Crocidosema* sp. | 2 | H6(2) |
| Foz do Jordão | PR | -25.6861 | -52.1146 | SU 3 | *Crocidosema* sp. | 1 | H6 |
| Londrina | PR | -23.1806 | -51.1718 | SE 2 | *Crocidosema* sp. | 1 | H21 |
| Manoel Ribas | PR | -24.4188 | -51.6651 | SE 2 | *Crocidosema* sp. | 3 | H1(2), H4 |
| Manoel Ribas | PR | -24.4099 | -51.6640 | SE 2 | *Crocidosema* sp. | 2 | H1, H8 |
| Maringá | PR | -23.5170 | -52.0266 |  | *A. sphaleropa* | 1 |  |
| Piraí do Sul | PR | -24.5601 | -49.9295 |  | *A. sphaleropa* | 1 |  |
| Piraí do Sul | PR | -24.5601 | -49.9295 | SE 1 | *Crocidosema* sp. | 1 | H16 |
| Piraí do Sul | PR | -24.5425 | -50.0114 |  | *A. sphaleropa* | 1 |  |
| Pitanga | PR | -24.7161 | -51.7700 | SE 2 | *Crocidosema* sp. | 1 | H22 |
| Reserva | PR | -24.6980 | -50.8999 | SE 1 | *Crocidosema* sp. | 3 | H1, H5, H15 |
| Tibagi* | PR | -24.4399 | -50.2693 | SE 1 | *Crocidosema* sp. | 3 | H6(3) |
| Capão Bonito do Sul* | RS | -28.1015 | -51.4854 | SU 1 | *Crocidosema* sp. | 3 | H6, H7, H25 |
| Colorado* | RS | -28.3948 | -52.9703 | SU 2 | *Crocidosema* sp. | 7 | H1, H5, H6(2), H8, H17, H23 |
| Coxilha | RS | -28.0990 | -52.3392 | SU 2 | *Crocidosema* sp. | 1 | H6 |
| Lagoa Vermelha | RS | -28.1074 | -51.5270 | SU 1 | *Crocidosema* sp. | 2 | H15, H17 |
| Passo Fundo | RS | -28.1584 | -52.5422 | SU 2 | *Crocidosema* sp. | 5 | H6, H26, H27, H28, H29 |
| Sananduva | RS | -27.9313 | -51.7982 | SU 1 | *Crocidosema* sp. | 3 | H6(2), H19 |
| Sarandi | RS | -27.7735 | -52.8180 |  | *A. sphaleropa* | 2 |  |
| Vacaria | RS | -28.5866 | -51.0289 | SU 1 | *Crocidosema* sp. | 1 | H6 |
| Vila Langaro | RS | -28.1352 | -52.1580 | SU 2 | *Crocidosema* sp. | 5 | H1, H6, H8, H10, H19 |
| Campos Novos | SC | -27.3675 | -51.2459 | SU 3 | *Crocidosema* sp. | 2 | H5, H6 |
| Chapecó | SC | -27.1112 | -52.7598 |  | *A. sphaleropa* | 2 |  |
| Chapecó | SC | -27.1112 | -52.7598 | SU 3 | *Crocidosema* sp. | 2 | H1, H6 |
| Guatambú | SC | -27.1112 | -52.7598 | SU 3 | *Crocidosema* sp. | 1 | H8 |
| Xanxerê | SC | -26.8473 | -52.3979 | SU 3 | *Crocidosema* sp. | 4 | H1(2), H20, H21 |
| Buri | SP | -23.7950 | -48.4297 | SE1 | *Crocidosema* sp. | 1 | H6 |
| Casa Branca | SP | -21.9077 | -47.2096 |  | *A. sphaleropa* | 3 |  |
| Itaí | SP | -23.4146 | -49.0842 | SE1 | *Crocidosema* sp. | 4 | H6(2), H30, H31 |
| Itapetininga | SP | -23.6149 | -48.0121 | SE1 | *Crocidosema* sp. | 1 | H18 |
| Itararé* | SP | -24.0280 | -49.3590 | SE1 | *Crocidosema* sp. | 3 | H6(2), H7 |

* Adults, which larvae were kept in artificial diet until adult phase. SU (South), SE (Southeast), CO (Middle west)


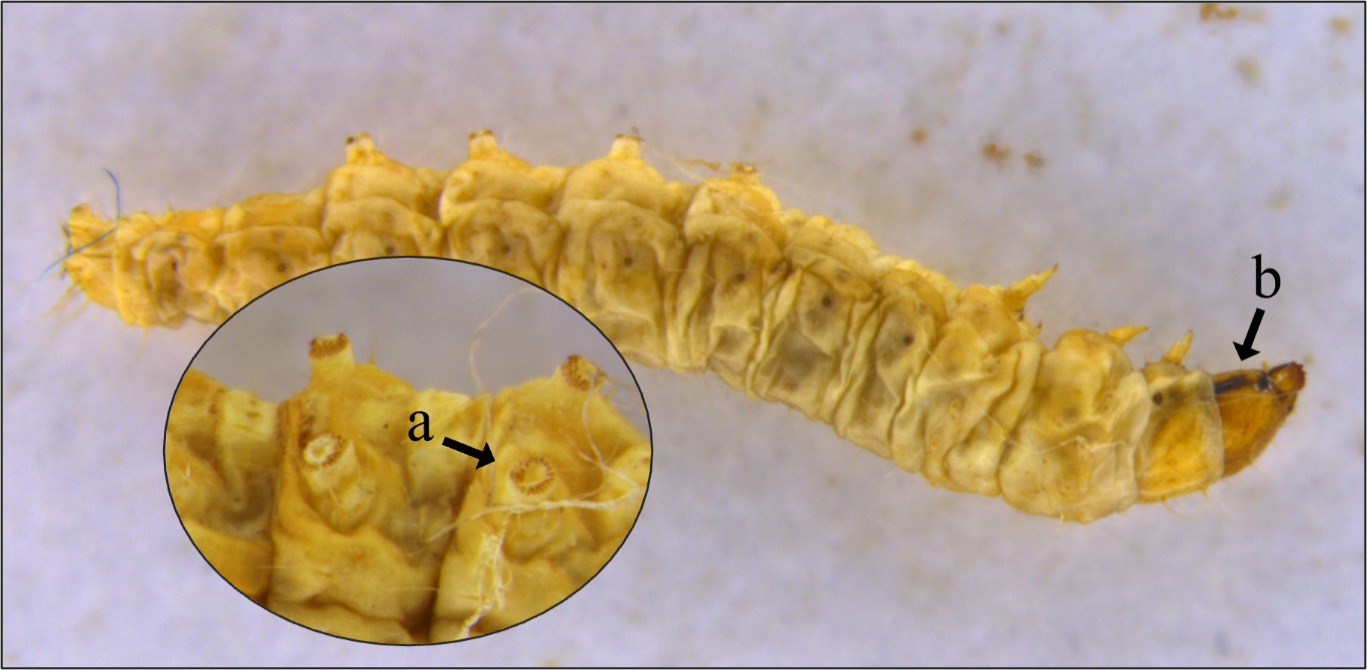


**Figure S1.** Image of *Crocidosema* sp. larvae, in lateral view, thirty to forty biordinal crochets in abdominal prolegs (a), head extending into the first segment of the thorax, lateral spot line in head extending from postgenal suture (b).


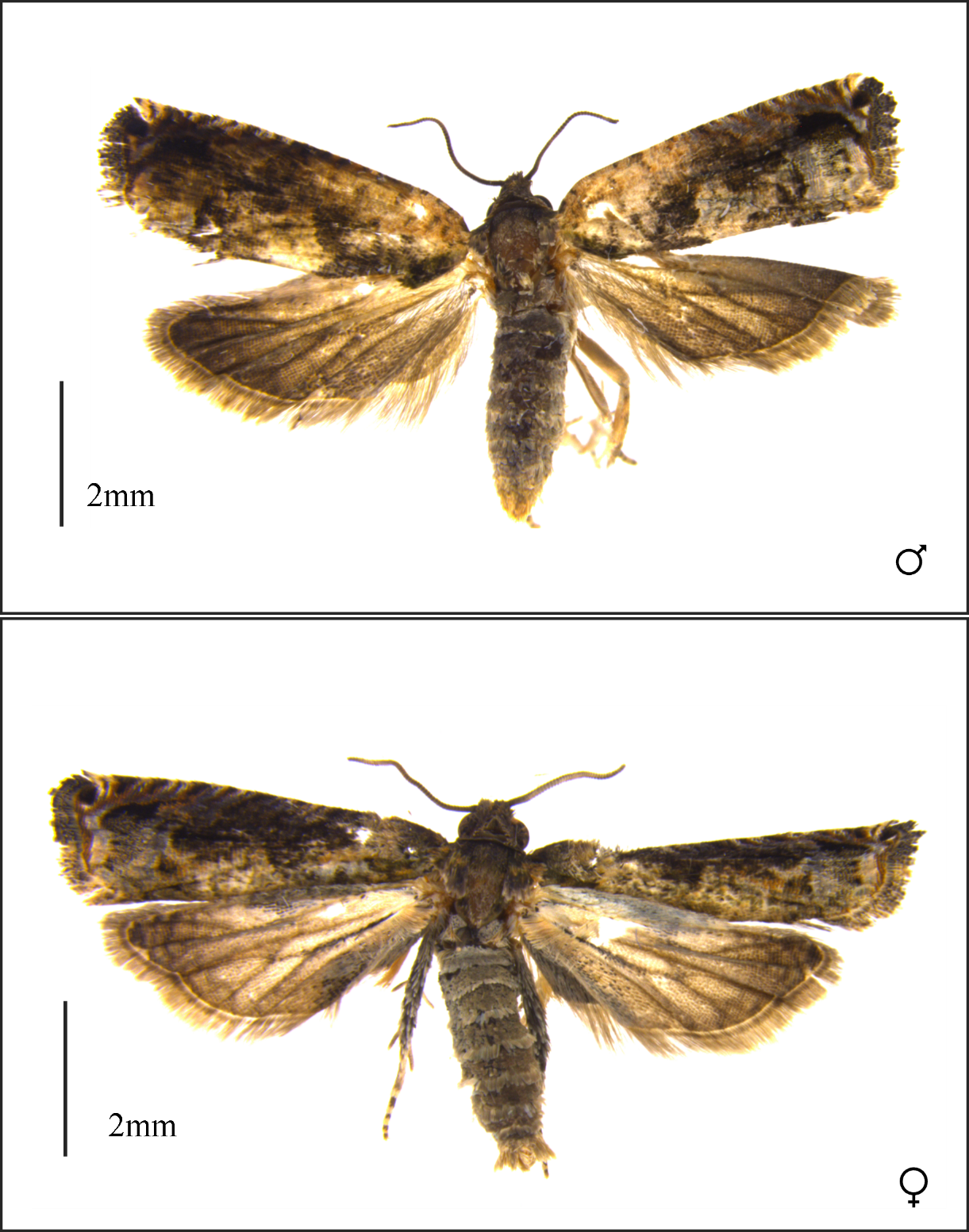


**Figure S2.** Image of Brazilian *Crocidosema* sp. adults (male and female) collected on soybean fields as larvae and reared in the laboratory until adult.

.


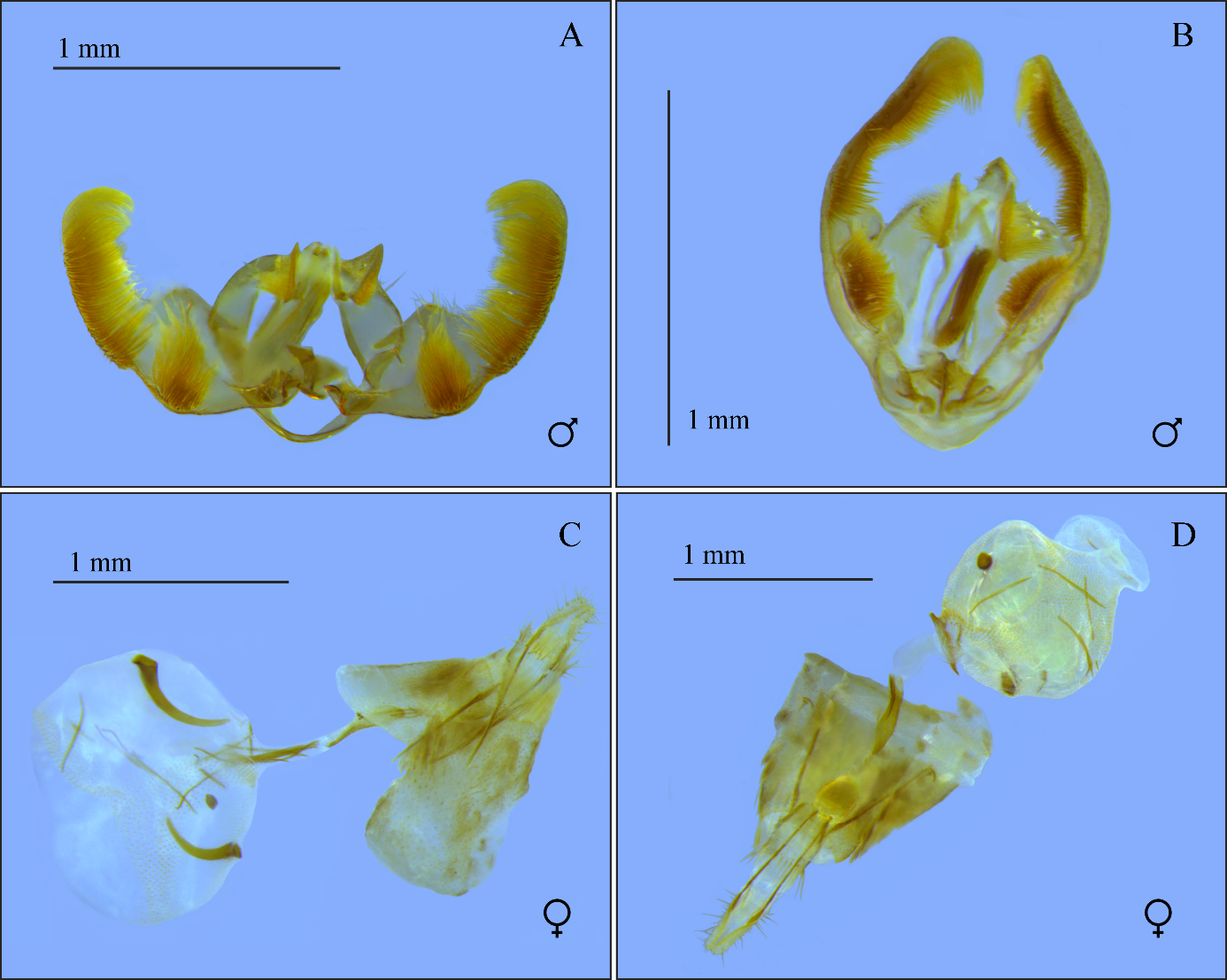


**Figure S3.** Image of Brazilian *Crocidosema* sp. genitalia collected in Capão Bonito do Sul – RS (Brazil) in soybean fields as larvae and reared in the laboratory. Male genitalia (A, B) with short triangular uncus, large triangular socii, parallel-sided cucullus with lot of bristles and edeagous with cornutii. Female genitalia (C, D) with pair of rounded pockets on sternum VII, characteristic apophysis, characteristic ostium bursae, and bursa copulatrix with a pair of signa.

**Table S2**. Haplotypes OTUs and BOLD codes of Crocidosema sequences used for genetic distance calculation and Bayesian phylogenetic estimation.

| **Species** | **BOLD CODE** | **Species** | **BOLD CODE** |
| --- | --- | --- | --- |
| C.accessa_ARG1 | IPTL118-13 | C.plebejana_GER1 | LOCBD841-06 |
| C.accessa_ARG2 | GMAFA348-15 | C.plebejana_USA2 | LNAUT1863-14 |
| C.lantana_CRI1 | ASHYC5522-12 | C.plebejana_SAF1 | LNAUT1864-15 |
| C.lantana_CRI2 | BLAPAA13535-18 | C.plebejana_SAF2 | LNAUT1864-16 |
| C.lantana_CRI3 | PLAES040-21 | C.plebejana_AUS2 | LCANA070-06 |
| C.lantana_CRI4 | PLXCQ873-20 | C.polyphae | ANICW737-11 |
| C.lantana_IND1 | GBMNE63102-22 | C.aporema_CRI1 | BLPAA1760-17 |
| C.lantana_IND2 | GBMNE63102-23 | C.aporema_CRI2 | BLPAA2378-17 |
| C.lantana_AUS1 | IMLS155-12 | C.aporema_CRI3 | BLPAA8017-17 |
| C.lantana_JAM1 | ITLP113-13 | C.aporema_CRI4 | BLPDF2779-19 |
| C.lantana_AUS3 | LOQTI443-11 | C.aporema_CRI5 | BLPDM2051-10 |
| C.lantana_MDG1 | MIMAD206-15 | C.aporema_CRI6 | BLPDT304-10 |
| C.litchivora1 | CCDB-2076-A04 | C.aporema_CRI7 | BLPDT304-10 |
| C.litchivora2 | CCDB-2076-A05 | C.aporema_CRI8 | BLPED974-12 |
| C.litchivora3 | ITLP117-13 | C.aporema_CRI9 | BLPEF3640-13 |
| C.longipalpana1 | GMPRC022 | C.aporema_CRI10 | BLPEF4364-13 |
| C.longipalpana2 | GMPRC068-16 | C.aporema_CRI11 | BLPEF9856-13 |
| C.perplexana1 | LNAUV874-17 | C.aporema_CRI12 | BLPEG1222-14 |
| C.perplexana2 | LNAUV875-17 | C.aporema_CRI13 | MHMYK14478-17 |
| C.plebejana_AUS1 | BBLOC731-11 | C.aporema_CRI14 | MHMYK14479-16 |
| C.plebejana_USA1 | BLPEE1830-12 | C.aporema_CRI15 | BLPAA2374-17 |
| C.plebejana_CRI1 | BLPEG1126-14 | C.aporema_CRI16 | BLPDT1207-10 |
| C.plebejana_CRI2 | FBLMS330-09 | C.aporema_CRI17 | BLPEF6658-14 |
| C.plebejana_CRI3 | LOCBF4988-15 | C.aporema_CRI18 | BLPEF6763-14 |
